# DAMPs from Cell Death to New Life

**DOI:** 10.3389/fimmu.2015.00422

**Published:** 2015-08-18

**Authors:** Emilie Vénéreau, Chiara Ceriotti, Marco Emilio Bianchi

**Affiliations:** ^1^Chromatin Dynamics Unit, Division of Genetics and Cell Biology, San Raffaele Scientific Institute, Milan, Italy; ^2^HMGBiotech Srl, Milan, Italy; ^3^Università Vita-Salute San Raffaele, Milan, Italy

**Keywords:** DAMP, tissue repair, HMGB1, ATP, inflammation

## Abstract

Our body handles tissue damage by activating the immune system in response to intracellular molecules released by injured tissues [damage-associated molecular patterns (DAMPs)], in a similar way as it detects molecular motifs conserved in pathogens (pathogen-associated molecular patterns). DAMPs are molecules that have a physiological role inside the cell, but acquire additional functions when they are exposed to the extracellular environment: they alert the body about danger, stimulate an inflammatory response, and finally promote the regeneration process. Beside their passive release by dead cells, some DAMPs can be secreted or exposed by living cells undergoing a life-threatening stress. DAMPs have been linked to inflammation and related disorders: hence, inhibition of DAMP-mediated inflammatory responses is a promising strategy to improve the clinical management of infection- and injury-elicited inflammatory diseases. However, it is important to consider that DAMPs are not only danger signals but also central players in tissue repair. Indeed, some DAMPs have been studied for their role in tissue healing after sterile or infection-associated inflammation. This review is focused on two exemplary DAMPs, HMGB1 and adenosine triphosphate, and their contribution to both inflammation and tissue repair.

## Introduction

Our body evolved mechanisms to detect pathogens through the recognition of conserved molecular motifs, called pathogen-associated molecular patterns (PAMPs). The binding of these molecules to pattern recognition receptors (PRR), such as Toll-like receptors (TLR), triggers the response of the immune system against the intruder (1). However, this “Stranger Theory” could not explain why strong immune responses are elicited in sterile conditions such as ischemic injuries, trauma, tumors, tissue transplants, and autoimmune diseases. By symmetry to the PAMP concept, Polly Matzinger proposed the “Danger Theory” in which the injured tissues were postulated to release intracellular molecules [damage-associated molecular patterns (DAMPs)] that activate the immune system (2). This concept has roots in a clinical trial on kidney transplantation, in which the oxygen free radical scavenger superoxide dismutase was exploited to avoid reperfusion injury (3). However, for many years the “Danger Theory” remained a theoretical model, until High Mobility Group Box 1 (HMGB1) and uric acid crystals were recognized as DAMPs (4, 5). Since then, many more DAMPs were identified and their roles in health and disease are now partially understood (Table 1).

**Table 1:** **List of putative DAMPs and role in inflammation and tissue repair**.

	DAMPs	Receptors	Release	Role in inflammation/immunity	Role in tissue repair	Reference
Nucleus	Histones	TLR2, TLR4 and TLR9	P, S and A	TLR- and inflammasome-dependent inflammatory response	N.D.	(6)
	Genomic DNA	TLR9	P	TLR9- and NALP3-mediated innate immune response, DC maturation	N.D.	(6)
	HMGB1	TLR2, TLR4, RAGE and TIM3	P and A	Recruitment/activation of immune cells	Migration/proliferation of stem cells, pro-angiogenic mediator.	(7)
	IL1a	IL-1R	P	Strong pro-inflammatory activity	Protective during early phase of inflammation	(7)
	IL33	ST2	P	Secretion of pro-inflammatory and Th2 cytokines	Epithelial cells proliferation and mucus production in the gut	(8)
Cytosol	ATP	P2Y2 and P2X7	P and A	Macrophages recruitment, IL-1β production by DC, antitumor immunity	Migration/proliferation of epithelial and endothelial cells, pro-angiogenic role	(9)
	F-actin	DNGR1	P	Contribution in recognition of necrotic cells by DC	N.D.	(10)
	Cyclophilin A	CD147	A	Inflammatory cells recruitment, inflammatory mediators release	N.D.	(10)
	HSPs	CD91, TLR2, TLR4, SREC1 and FEEL1	P, S and A	Recruitment of immune cells DC maturation, T cell-based antitumor immunity	Wound debris clearance, cell migration/proliferation and collagen synthesis in skin	(7)
	Uric acid crystals	NLRP3	P	DC maturation and neutrophil recruitment	N.D.	(7)
	S100s	TLR2, TLR4, RAGE	P	Potent immunostimulatory activity, monocytes and neutrophils recruitment	Myoblast proliferation/differentiation	(7)
Mitochondria	Mitochondrial DNA	TLR9	P	Macrophages and neutrophils activation	N.D.	(11)
	Mitochondrial trascription factor A	RAGE and TLR9	P	DC activation, type I interferon release	N.D.	(11)
ER	Calreticulin	CD91	P and S	Potent “eat me” signal, mediator of tumor immunogenicity	Cell migration/proliferation, extracellular matrix production	(10)

*P, passive release; A, active secretion; S, surface exposure; ER, endoplasmic reticulum; N.D. not described*.

Damage-associated molecular patterns are molecules that have a physiological “day-time job” inside the cell, and have the additional job of signaling cell damage when they are outside the cell. Location, inside vs. outside the cells, is critical: DAMPs are invisible to the immune system when performing their day-time job, and become visible only when exposed to the extracellular environment.

Timing is also important. Initially, DAMPs were expected to attest cell death, and therefore to be released passively from dead cells. Indeed, HMGB1 was identified as a DAMP because it is passively released by necrotic cells, which undergo an untimely death, but not by apoptotic cells, which eliminate themselves in an elaborately programed way (4). However, an important addition to the DAMP concept is that DAMPs do not necessarily originate from dead cells: DAMPs can be secreted or exposed by living cells undergoing a life-threatening stress. Indeed, alerting the immune system as soon as possible can bring advantages. HMGB1 can be secreted by stressed cells via a private secretion pathway, not involving the endoplasmic reticulum (12, 13). Adenosine triphosphate (ATP) can be actively released via vesicles and connexin or pannexin hemichannels (14). Other DAMPs, such as calreticulin and heat shock protein 90 (HSP90), are exposed *de novo* or become enriched on the outer leaflet of the plasma membrane (15).

It is now time to recognize another essential feature of DAMPs: they are essential for tissue healing after inflammation, both sterile and infection-associated. This review will focus on two exemplary DAMPs, HMGB1 and ATP, and their contribution to both inflammation and tissue repair.

## HMGB1 and ATP as Exemplary DAMPs

### HMGB1, a redox-sensitive DAMP

HMGB1 is a mobile chromatin protein that acts as a DNA chaperone, by binding DNA transiently and bending it reversibly. As a DNA chaperone, it facilitates nucleosome formation, contributes to the binding of proteins, including transcription factors that distort DNA upon binding, and participates in transcription, replication, and DNA repair (16). HMGB1 is constitutively expressed in almost all cell types, and to act as a DAMP it must relocate into the external environment: it is passively released following traumatic cell death (but not apoptosis) and is secreted during severe stress (4, 17).

HMGB1 secretion is not completely understood. Drawing a comparison with another leaderless protein, IL-1β, a “two-step model” for HMGB1 secretion was proposed, which involves a first trigger to induce HMGB1 acetylation and cytoplasmic translocation and a second trigger to elicit its extracellular transport (18) Indeed, secreted HMGB1 (as opposed to HMGB1 passively released by dead cells) is hyperacetylated (19). In accordance with the two-step model, Lu et al. (20) have demonstrated that the inflammasome, in particular NLRP3, is involved in the release of HMGB1. Inflammasomes are large caspase-1-activating complexes, composed by the assembly of proteins that are ultimately activated by both PAMPs and DAMPs (21). There are multiple inflammasome complexes, and among them the one containing NLRP3 (also known as NALP3 and cryopyrin) is the most studied. Since the synthesis of NLRP3 is triggered by TLR signaling, it has recently been proposed that HMGB1 itself could “prime” the inflammasome through its binding to TLR2/TLR4 (22). Indeed, the role of HMGB1 in inflammasome activation has been demonstrated in a model of heatstroke-induced liver injury (23).

Once in the extracellular milieu, HMGB1 signals danger to the surrounding cells, triggers inflammation, and activates innate and adaptive immunity by interacting with multiple receptors (24).

The first receptor described for HMGB1 is the receptor for advanced glycation endproducts (RAGE), a multifunctional transmembrane protein of the immunoglobulin superfamily (25). Under physiological conditions, RAGE is expressed at low levels in the majority of tissues and, interestingly, at high levels in the lung. In pathophysiological conditions such as chronic inflammation, RAGE expression is considerably increased in different tissues, in particular activated endothelium and leukocytes (26). HMGB1 signaling through RAGE leads to activation of the nuclear factor-κB (NF-κB) pathway, as well as to signal transduction through JNK, and p38 (27). In addition, HMGB1/RAGE interactions lead to the activation of the ERK MAP kinase pathway, which is important in cell migration, tumor proliferation and invasion, and expression of matrix metalloproteinases. The HMGB1/RAGE axis is mainly involved in the recruitment and migration of cells, directly by inducing expression of adhesion molecules, such as VCAM-1 and ICAM-1 (28), or indirectly by inducing secretion of chemokines, in particular CXCL12, which in turn forms a heterocomplex with HMGB1 (29).

HMGB1 also binds to TLRs. In complex with CpG-ODNs, HMGB1 binds to TLR9 and enhances cytokine production in plasmacytoid dendritic cells (DCs) (30). When HMGB1 is bound to nucleosomes, it activates macrophages and DCs through TLR2 (31). However, most studies focused on the HMGB1/TLR4 axis. TLR4 mediates cell responses to lipopolysaccharide (LPS), but responds to several DAMPs as well. The contribution of the HMGB1/TLR4 axis to inflammation and immune regulation has been demonstrated in a wide range of experimental models, such as liver and lung damage, cancer, and epilepsy (32–35). Recently, a large body of evidence demonstrated that the redox state of cysteines modulates the binding of HMGB1 to its receptors, and consequently its activities.

**Figure 1:**
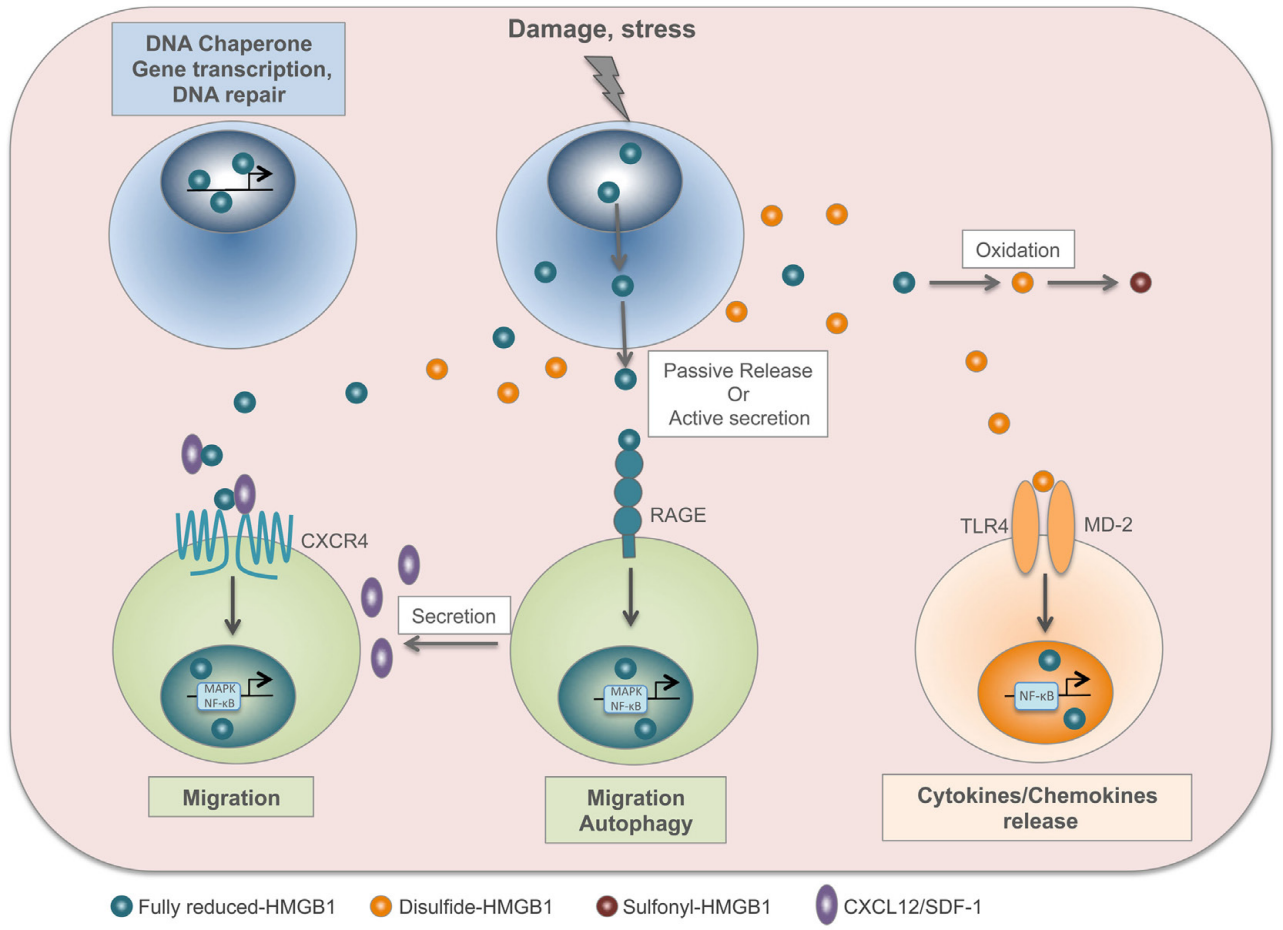
**HMGB1 is a redox-sensitive DAMP**. In the nucleus, fully reduced HMGB1 acts as a DNA chaperone and contributes to gene transcription and DNA repair. Upon injury or stress, HMGB1 is passively released by dead cells or actively secreted by stressed cells. The fully reduced HMGB1 binds to CXCL12 chemokine to form a heterocomplex, which in turn binds to CXCR4 and induces cell migration. In addition, HMGB1 interacts with RAGE to induce CXCL12 secretion and autophagy. In the extracellular compartment, disulfide HMGB1 derives from the active secretion and/or the conversion of fully reduced HMGB1 by oxidation. Disulfide HMGB1 binds to TLR4/MD-2 complex and induces cytokine/chemokine release. Finally, HMGB1 cysteines are terminally oxidized to sulfonates; sulfonyl-HMGB1 is neither chemoattractant nor has cytokine-inducing activity.

HMGB1 contains three cysteines: C23 and C45 can form a disulfide bond, and C106 is unpaired. These cysteines are modified by redox reactions, giving rise to three isoforms named “fully reduced HMGB1” for the all-thiol form, “disulfide HMGB1” for the partially oxidized one, and “sulfonyl HMGB1” for the terminally oxidized form (36). Fully reduced HMGB1 forms a heterocomplex with the chemokine CXCL12, which binds with increased affinity to its CXCR4 receptor (29). Conversely, the extracellular TLR4 adaptor myeloid differentiation factor 2 (MD-2) binds specifically to disulfide HMGB1, and not to the other redox forms, triggering the expression of chemokines and cytokines (37). Notably, interaction with MD-2 also requires the third cysteine, in the fully reduced form. Thus, the disulfide bond between C23 and C45 makes HMGB1 a proinflammatory cytokine, whereas further cysteine oxidation to sulfonates abrogates both the chemoattractant and proinflammatory activities of HMGB1 (38). Several studies demonstrated a correlation between the presence of the disulfide HMGB1 and the onset of pathologies such as brain injury, liver damage, myositis, and juvenile idiopathic arthritis (19, 39–41). Moreover, disulfide HMGB1, and not the reduced form, contributes to nociceptive signal transmission via activation of TLR4 (42) (Figure 1).

The HMGB1 inside the cell (nucleus or cytosol) is completely reduced, and early prevalence of fully reduced HMGB1 and subsequenct appearance of disulfide HMGB1 were observed in models of brain, muscle, or liver injuries and in patients with Juvenile Idiopathic Arthritis (19, 39, 41). Supernatants from LPS-activated THP-1 monocytic cells contain both fully reduced and disulfide HMGB1 (38), suggesting that activated monocytes/macrophages contribute to inflammation by producing disulfide HMGB1. Tandem mass-spectrometric analysis showed that systemic levels of the disulfide HMGB1 isoform dramatically increased during early Macrophages Activation Syndrome (43). Similarly, a study revealed that cells undergoing unprimed pyroptosis release a reduced HMGB1 redox isoform, whereas priming with TLRs ligands results in the conversion to disulfide HMGB1 (44).

In conclusion, it is now essential to identify the redox state of HMGB1 in each specific condition and locale *in vivo*.

### ATP, a time-resolved DAMP

Nucleotides as well, particularly ATP, have both intra- and extracellular roles. They are well known for their function as a universal energy source in cell reactions and metabolism. The multiple functions of extracellular ATP have been known since the late 1940s, when its vasoactive property and its release in shock were discovered [reviewed by Gordon (45)]. Later, ATP was found to be released at nerve terminals, affecting smooth muscle tone. Moreover, ATP and adenosine are involved in the mechanisms underlying local control of vessel tone, while ADP induces platelet aggregation and is released, together with ATP, from platelet granules (45). Several cell types release ATP during inflammatory, ischemic, and hypoxic conditions. ATP release can occur in a passive fashion, for example during necrosis, but many molecular pathways have been described for active release, as ATP-containing lysosome exocytosis from astrocytes, pannexin-mediated ATP release during apoptosis, and connexin- or pannexin-mediated ATP release from inflammatory cells, such as neutrophils (46). Moreover, it has been recently demonstrated that ATP can also be secreted by dying cancer cells through the classical endoplasmic reticulum/Golgi secretory pathway (47).

In the extracellular compartment, nucleotide signaling is intrinsically short-lived. Signaling is terminated in the timescale from seconds to minutes by the enzymatic conversion of ATP to adenosine through the ecto-nucleoside triphosphate diphosphohydrolase CD39 (from ATP/ADP to AMP) and the ecto-5′-nucleotidase CD73 (from AMP to adenosine) (48). ATP acts as a signaling molecule through the activation of purinergic P2 receptors (9). These receptors have a widespread expression throughout different tissues and are involved in innate and adaptive immune responses (46, 48). P2 receptors can be further subdivided into metabotropic P2Y receptors (P2YRs), which are G-protein-coupled, and ionotropic P2X receptors (P2XRs), which are nucleotide-gated ion channels.

P2YR signaling has been linked with chronic inflammation, and one of the most studied receptor of this class is P2Y2R, which is activated by UTP or ATP. P2Y2R agonists promote mucociliary clearance and wound healing [reviewed by Idzko et al. (9)]. For these reasons, P2YR agonists were exploited for the treatment of cystic fibrosis (49). Apoptotic cells release ATP as a “find-me” signal that binds P2Y2R on macrophages, stimulating their phagocytic activity and the clearance of apoptotic cells (50). During pneumonia, neutrophil-dependent ATP release and autocrine activation of P2Y2R contribute to purinergic chemotaxis, thereby enhancing bacterial clearance (51). However, ATP-elicited P2Y2R signaling can lead to uncontrolled inflammation and chronic inflammatory diseases. On alveolar epithelial cells or eosinophils, P2Y2R signaling causes production of pro-allergic mediators (for example, IL-33, IL-8, eosinophil cationic protein) during allergic airway disease (52). Similarly, P2Y2R signaling on DC has a role during the induction and self-perpetuation of asthma (53). In general, P2Y2R antagonists can evolve into useful drugs for chronic inflammatory diseases.

**Figure 2:**
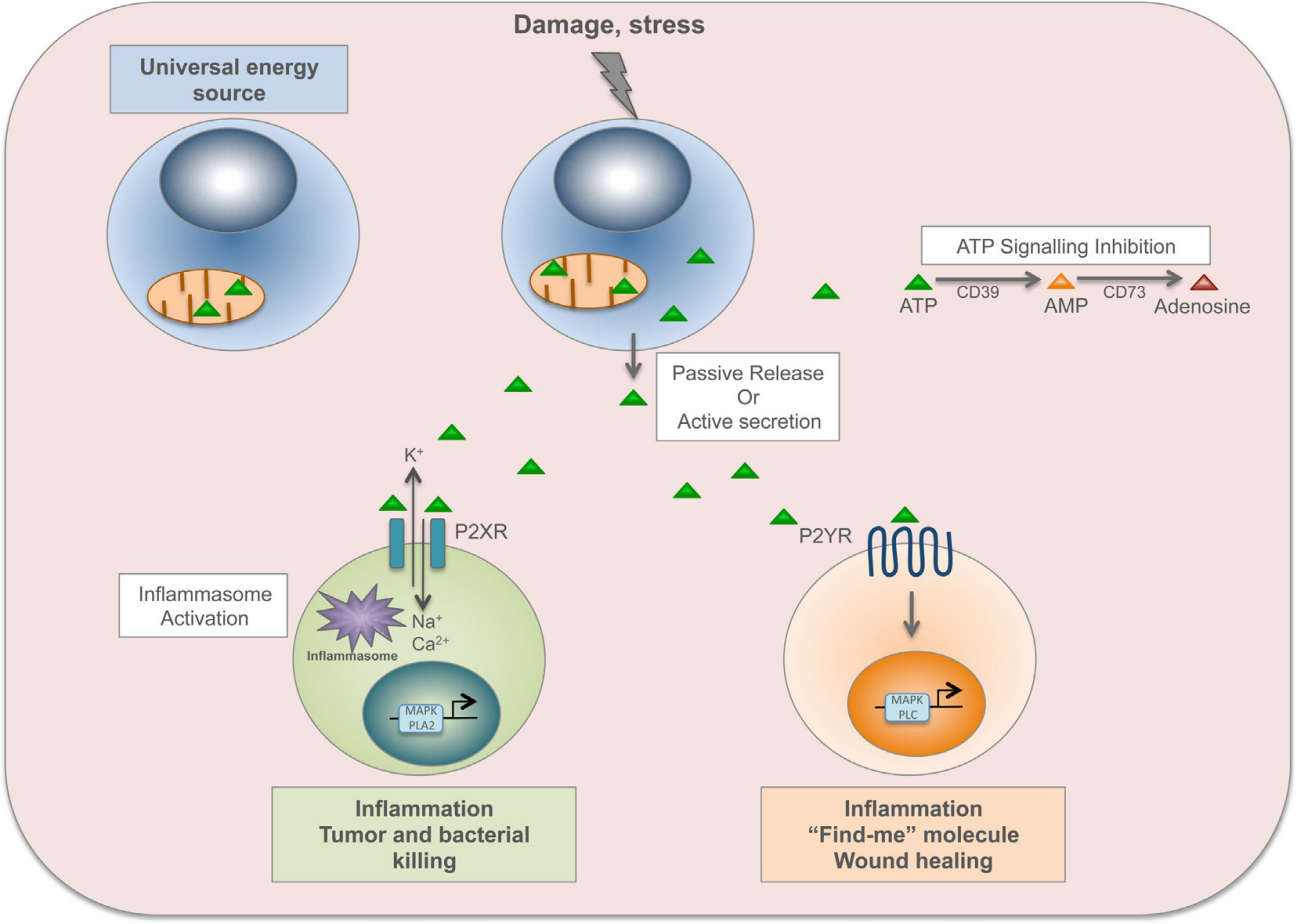
**ATP is a time-resolved DAMP**. In the cell, ATP derived from mitochondria is a universal energy source in cell reactions and metabolism. Upon damage or stress, ATP, and other nucleotides, are passively released by dead cells or actively secreted by stressed cells. ATP binds to ionotropic P2X receptors (P2XR), which are nucleotide-gated ion channels, allowing sodium (Na^+^) and calcium (Ca^2+^) influx and potassium (K^+^) efflux. The increased level of intracellular calcium activates p38 MAPK or phospholipase A2 signaling, while potassium efflux activates the inflammasome. P2XR signaling is involved in inflammation, tumor and bacterial killing. ATP also binds to metabotropic P2Y receptors (P2YR), which are G-protein-coupled, and induces activation of MAPK and phospholipase C (PLC). P2YR signaling is implicated in inflammation and wound healing, and ATP released by apoptotic cells acts as a “find-me” signal to recruit macrophages to the site of damage and to promote clearance of apoptotic cells. ATP signaling is abolished by the enzymatic conversion of ATP to adenosine through the ecto-nucleoside triphosphate diphosphohydrolase CD39 (from ATP to AMP) and the ecto-5′-nucleotidase CD73 (from AMP to adenosine).

P2XR channels are opened by the binding of ATP, allowing sodium and calcium influx and potassium efflux. The increased level of intracellular calcium activates p38 MAPK or phospholipase A2 signaling, while potassium efflux activates the inflammasome (9). Then, P2XR channels gradually dilate into pores permeable to larger organic cations and small hydrophilic molecules with a molecular mass below 900 dalton (including ATP) (54). Among P2XRs, P2X7R is predominantly expressed on immune cells such as mast cells, macrophages, microglia, and DCs, and its signaling has been linked to inflammatory and infectious disorders (46). P2X7R is required for appropriate inflammatory defense mechanism against invading pathogens and cancer cells. For instance, it is important during intracellular killing of *Mycobacterium tuberculosis* by macrophages (55). Dying tumor cells release ATP that activates P2X7R on DCs, which in turn promote the priming of IFN-γ-producing cytotoxic CD8^+^ T cells that kill cancer cells (56). On the other hand, P2X7R signaling contributes to the induction and maintenance of chronic inflammation. Indeed, P2X7R signaling on DCs is involved in the sensitization phase of allergic disorders such as contact hypersensitivity (through CD81 T-cell priming) (57) and asthma (through CD41 T-cells, TH2 response) (58), and contributes to transplant rejection (through CD41 T cells, TH1 response) (59). Furthermore, P2X7R signaling on enteric neurons or mast cells has been implicated in promoting intestinal inflammation during inflammatory bowel disease (60) (Figure 2).

As already mentioned, the binding of extracellular ATP to P2X7R elicits NLRP3 activation (21). The contribution of the ATP/P2X7 receptor axis to inflammasome activation in pathogenic conditions has been shown in a bleomycin model of pulmonary inflammation in mice. This leads to IL-1β maturation and secretion, causing lung inflammation that evolves to fibrosis (61). Moreover, P2X7R upregulation in atherosclerotic lesions in mice modulates NLRP3 inflammasome activation, and is involved in the progression and development of atherosclerosis (62).

As we reviewed in this chapter, DAMPs, in particular HMGB1 and ATP, have been linked to inflammation and related disorders. Hence, inhibition of DAMP-mediated inflammatory responses might appear as a promising strategy to improve the clinical management of infection- and injury-elicited inflammatory diseases. However, it is important to keep in mind that these sophisticated molecules are danger signals important not only for the inflammatory response but also for tissue repair. Here, we review the latest findings on the regenerative properties of HMGB1 and ATP.

## HMGB1 and ATP in Tissue Repair

**Figure 3:**
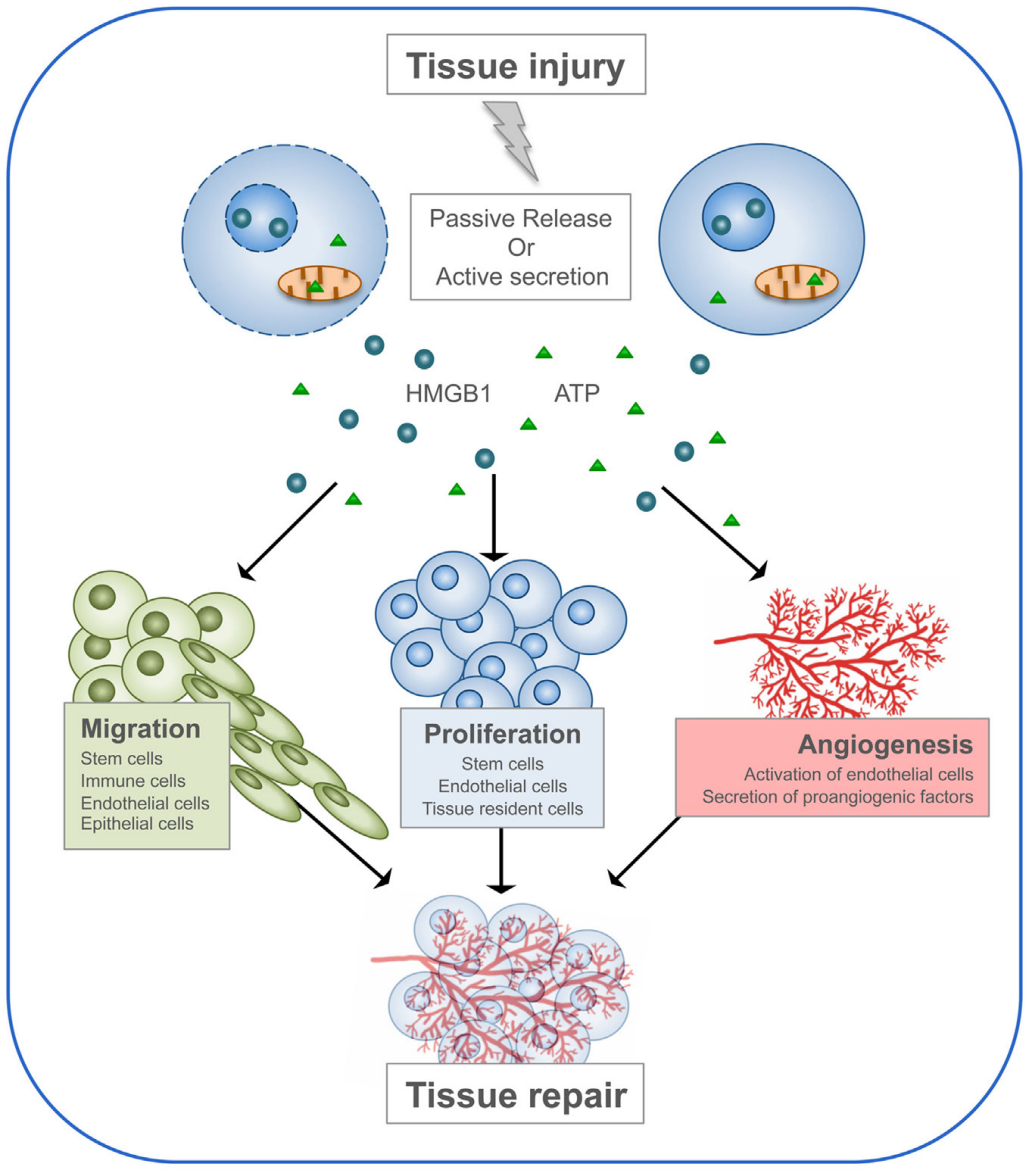
**HMGB1 and ATP in tissue repair**. Following tissue injury, HMGB1 and ATP are passively released by dead cells or actively secreted by stressed cells. Then, they recruit to the site of damage the cell types required to heal the wound. First, immune cells are needed to clean the wound by engulfing dead cells and cellular debris. Then, stem cells and neighboring cells are induced to proliferate and build new tissue, together with its extracellular matrix. Endothelial cells are activated to form new blood vessels.

The functions of DAMPs consist in alerting the body about danger, stimulating the immune system in order to initiate the immune response, and finally promoting the regeneration process. This last property of DAMPs has been particularly investigated for two members of the family: HMGB1 and ATP (Figure 3).

### HMGB1, a chemotactic and proangiogenic DAMP

HMGB1 plays an important role in promoting tissue regeneration after acute inflammation. Locally released HMGB1 recruits bone-marrow derived mesenchymal stem cells (MSCs), and promotes the proliferation and differentiation of tissue-associated resident stem cells, such as dental pulp stem cells, mesoangioblasts, and MSCs (63). Adult MSCs have attracted intense interest because they can be isolated from the bone marrow and can be expanded in culture while maintaining their multipotency, and thus may be used for the repair of bone, cartilage, muscle, bone marrow stroma, tendon, fat, and other connective tissues. HMGB1 induces migration of MSC (64–66) and their differentiation into osteoblasts (64). Moreover, intravenous administration of HMGB1 in mice induces MSC accumulation in skin grafts, promoting inflammatory suppression in the grafts, and subsequent tissue regeneration (67). However, a recent study showed that HMGB1 induces migration of monocytes but not of MSCs (68). Further experiments are necessary in order to understand these discrepancies, in particular, the culture conditions that could modulate the redox state of HMGB1 and consequently its chemotactic activity.

Tissue repair requires angiogenesis, and numerous studies have identified HMGB1 as a proangiogenic factor [recently reviewed by Yang et al. (69)]. Briefly, HMGB1 plays an important role in neovascularization of ischemic areas by recruiting endothelial progenitor cells through activation of integrins and inducing the migration and sprouting of endothelial cells in a RAGE-dependent manner (70, 71). In addition, HMGB1 stimulates endothelial cells and macrophages to release proangiogenic cytokines, such as VEGF, TNF-α, and IL-8 (72). HMGB1 secreted by leukocytes is important for the skeletal muscle to react to hypoxia and to initiate angiogenesis in response to injury (73).

The regenerative properties of HMGB1 have been studied in different models of tissue injury, including spinal cord, skin, muscle, and heart. In a model of spinal cord injury in zebrafish, the authors observed that HMGB1 expression increases after injury in both motoneurons and endothelial cells. Moreover, inhibition of HMGB1 decreases locomotor recovery and axonal formation (74). In a model of spontaneous spinal cord regeneration in the gecko, HMGB1 does not mediate the inflammatory response but promotes regeneration. Here, HMGB1 induces migration of oligodendrocytes by interacting with RAGE, but not TLRs (75).

In skin, HMGB1 was identified as a chemoattractant for bone marrow-derived epithelial progenitors, which contribute to epithelial regeneration (67). A well-known consequence of diabetes is impaired skin wound repair, and topical treatment with recombinant fully reduced HMGB1 accelerated wound healing in diabetic mice (76). Accordingly, HMGB1 levels are low in diabetic human and mouse skin, and inhibition of endogenous HMGB1 impaired wound healing in non-diabetic mice but had no effect in diabetic mice. Conversely, HMGB1 also plays a role in scar formation in fetal skin (77). Interestingly, the authors used a recombinant HMGB1 described to induce TNF release, suggesting that it corresponds to the disulfide form.

In a mouse model of acute myocardial infarction, overexpression of HMGB1 in cardiac cells or local administration of HMGB1 induced myocardial regeneration, restored cardiac function, and improved survival (78, 79). These effects were due to proliferation and differentiation of cardiac stem cells and induction of angiogenesis. Moreover, HMGB1 stimulates primary cardiac fibroblasts to exert a paracrine action on cardiac stem cells (80), and intramyocardial injection of HMGB1 improved global cardiac function by reducing fibrosis and cardiomyocyte hypertrophy (81). HMGB1 activates a number of genes involved in cardiac protection and regeneration, and Notch1 signaling plays a key role in HMGB1 ability to activate cardiac stem cells (82). Interestingly, beneficial effects of HMGB1 were also observed in models of heart failure (81–83). Conversely, HMGB1 blockade caused an expansion of the infarct scar and marked hypertrophy of the non-infarcted area (84).

Finally, HMGB1 is important in skeletal muscle regeneration. The presence of only half of the normal amount of HMGB1 results in defective myogenesis both during development and after acute injury (85). In particular, the absence of HMGB1 in leukocytes results in defective angiogenesis and a delay in muscle regeneration (73). HMGB1 levels are increased in regenerating skeletal muscle after ischemia/reperfusion, and intramuscular administration of HMGB1 enhances both vascularization and myofiber formation (86). Besides angiogenesis, HMGB1 also promotes myogenesis by stimulating migration and proliferation of mesoangioblasts and migration of skeletal myoblasts and smooth muscle cells, and by accelerating myogenic differentiation (86–89).

In conclusion, HMGB1 released by injured tissues promotes tissue repair by inducing migration and proliferation of stem cells, and by promoting angiogenesis. However, several studies have demonstrated the beneficial effect of blocking HMGB1 in animal models of spinal cord, liver, brain, and myocardial damage after ischemia/reperfusion injury (24). Indeed, HMGB1 also activates fibroblasts and astrocytes, which might induce fibrosis as a program of tissue consolidation if successful regeneration is not achieved. The discrepancy between these results might be due to the fact that the redox state of HMGB1 has not been rigorously identified in most of the studies reported. Indeed, even if the fully reduced HMGB1 is the most used recombinant form, the reduced and disulfide forms can easily interconvert both *in vitro* and *in vivo*.

### Nucleotides as “find-me” signals in tissue repair

The interplay between nucleotides and the immune system is essential for regenerative processes in the body (46, 90). During tissue regeneration, the organism needs to remove dead cells and debris to recruit various types of cells and to stimulate their proliferation in order to achieve wound closure. Nucleotides participate actively to these three phases by interacting with purinergic receptors on different cell types.

The two families of P2Rs appear to have separate roles: P2XRs are involved in defense mechanisms and cell death, and P2YRs in wound healing (9). Indeed, prevalently P2YRs have been studied in different models of tissue regeneration. Both ATP and UTP released by apoptotic cells in a caspase-1-dependent manner act as “find-me” signals that recruit macrophages through P2Y2R, and stimulate their phagocytic activity (50). Neutrophils release ATP that in turn recruits neutrophils, in a feed forward loop (51). In addition, both ATP and UTP promote migration of vascular smooth muscle cells through binding of P2Y2R to filamin A (91).

Stimulation of P2Y receptors has a mitogenic effect on multiple cell types, including brain capillary endothelial cells (92), cardiac endothelial cells (93), and fibroblasts (94). Non-hydrolyzable nucleotide analogs (e.g., ATPγS, ADPβS) strongly promote proliferation of HUVEC cells and of mammalian vascular smooth muscular cells (95). These observations strongly suggest that nucleotide might be proangiogenic factors important for tissue repair.

Nucleotide release from dying cells after acute kidney injury induces proliferation of neighboring tubular cells, thus promoting wound closure via the downstream activation of Akt (96). In the liver, ATP released after partial hepatectomy, both from hepatocytes and from Kupffer cells, contributes to liver regeneration by activating cell cycle progression in hepatocytes (97). Calcium waves elicited by ATP released from damaged cells are important in the developing brain of *Xenopus laevis*, where neural progenitor cells reorganize their cytoskeleton and activate the actomyosin contractile machinery to drive the expulsion of damaged cells into the brain ventricle. This represents a mechanism for rapid wound healing in the developing brain (98).

Shockwave treatment is a new technology used to treat chronic painful conditions of the musculoskeletal system. Shockwaves induce ATP release, which leads to Erk1/2 and p38 MAPK activation and cell proliferation, and increased wound healing in a rat model (99). During skin wound healing, extracellular nucleotides have a dual function: they inhibit keratinocyte motility and facilitate migration of other cell types (e.g., endothelial cells) (100, 101). Treatment of mouse ear wounds with Mg-ATP encapsulated in lipid vesicles (ATP-vesicles) induced macrophage accumulation, *in situ* proliferation and new tissue growth (102). ATP release from HaCaT keratinocytes caused the propagation of intercellular calcium waves from cells at the frontier facing the scar toward the cells in the rear, in a P2Y-receptor-dependent manner (103). Finally, the most striking evidence of P2YR signaling in tissue repair is the delay of wound healing observed in *P2y2r*^−/−^ mice (94).

In zebrafish larvae, when the tail fin is wounded, osmolarity differences between the interstitial fluid and the ambient water trigger ATP release, which initiates rapid wound closure through long-range activation of basal epithelial cell motility. In this case, P2Y2R is probably irrelevant, since the P2Y2R inhibitor suramin had little effect, even at high concentrations (104). Indeed, wound healing is known to involve other purinergic receptors. In cystic fibrosis, ATP release from epithelial cells activates P2RY11 on nearby epithelial cells, stimulating proliferation, migration and wound repair (105). P2X7 activation participates in angiogenesis and wound repair by promoting VEGF release from human monocytes (106). Moreover, P2X7 is necessary for timely healing of abrasion wounds and normal stromal collagen structure (107). Acute UV irradiation of keratinocytes causes ATP release that triggers P2X7R on skin-resident T cells and participates to DNA repair response essential for skin regeneration (108). Thus, even P2XRs might switch from their killing activity, opening pores on the plasma membrane that cause the cell to collapse, to a pro-regenerative function, helping tissue repair.

## Conclusion and Future Directions

Nature is remarkably conservative, in that it uses the same molecule over and over again to attain related goals (109). DAMPs are exemplary from this point of view, as they are (generally abundant) molecules that are involved in the everyday functioning of the cell, and double up as signals of cell damage when they are present outside of the cell. As it happens, this simple invention that allowed to discern damage (DAMP-out) from normality (DAMP-in), could be used further to better describe the nature of the damage, and to record its occurrence for future memory. Thus, after being released (either passively or actively), DAMPs act to:

(1) convey the message of danger to other cells,
(2) trigger inflammation and activate innate immunity to stop the damage,
(3) participate in cell–cell communication that instructs adaptive immunity, to help establish immunological memory,
(4) orchestrate tissue repair and healing.

Points (1) and (2) have been widely described (7, 110). The cooptation of DAMPs into the process of immunological memory (point 3) and the related process of Immunogenic Cell Death are the subject of other reviews in this Frontiers collection. Immunogenic cell death is a perfect example of interplay between several DAMPs to alert and activate the immune system.

Here, we have focused on the role played by ATP and HMGB1 in wound repair and tissue reconstruction. ATP and other nucleotides, and their purinergic receptors, have been known to participate in tissue repair since the late 1990s, even before they were recognized as DAMPs. Examples involving HMGB1 are now as numerous. However, a fundamental problem must be acknowledged: how can the organism use the same DAMPs to trigger inflammation and to orchestrate tissue repair, which should occur *after* resolution of inflammation? Here, we can only speculate, and perhaps suggest future avenues of research. Usually, a signal with two possible meanings must be disambiguated by either the state of the receiver or the context of the signaling. Thus, to disambiguate the DAMP in inflammation and tissue repair, cells would need two different receptors, on different cells or on the same cell but at different times. Perhaps relevant here is that RAGE, a receptor for HMGB1, is low at the beginning of inflammation and induced by it.

Context in signaling is easy to picture: contextuality is paramount in everyday human communication. In the case of inflammation and tissue repair, context is the co-presence of other ligand-receptor pairs in different situations, in addition to the DAMP and its receptor, so that cells are differently activated or polarized. In fact, inflammation creates a microenvironment that is acidic, oxidizing (rich in oxygen and ROS), and where the metabolism of inflammatory cells is shifted toward glycolysis, whereas tissue repair occurs in a microenvironment which is neutral, reducing, and where macrophage metabolism is shifted towards oxidative phosphorylation and fatty acid oxidation (111, 112).

Also notable is that tissue reconstruction and inflammation, or at least some aspects of both, occur simultaneously in chronically inflamed tissue. In situations like rheumatoid arthritis, where inflammation is rampant and the synovia grows exuberantly into a pannus, DAMPs might not be disambiguated, and might actually activate both programs at the same time. Not surprisingly, targeting DAMPs or their receptors during chronic inflammation is often beneficial. However, finely tuning might be better than blocking them altogether. Thus, better understanding of the activity and the interaction of cells in inflammation and tissue reconstruction, and of DAMP signaling, is key to control excessive inflammation, resolve chronic inflammation, and promote tissue repair and healing.

## Conflict of Interest Statement

The authors have no conflicting financial interests. However, Marco Emilio Bianchi is founder and part owner of HMGBiotech, a company that provides goods and services related to HMGB proteins, and Emilie Vénéreau was partially supported by HMGBiotech.
